# Effects of different types of exercise on hypertension in middle-aged and older adults: a network meta-analysis

**DOI:** 10.3389/fpubh.2023.1194124

**Published:** 2023-09-20

**Authors:** Wei Gao, Moran Lv, Tao Huang

**Affiliations:** ^1^School of Physical Education and Sport Science, Fujian Normal University, Fuzhou, China; ^2^Institute of Physical Education, Huanggang Normal University, Huanggang, China

**Keywords:** hypertension, aerobics, static exercise, middle-aged and older adults, types of exercise

## Abstract

**Objective:**

This study mainly used network meta-analysis to explore the effect of different types of exercise on hypertension in middle-aged and older adults.

**Methods:**

Several databases (e.g., PubMed, Embase, and the Cochrane Library) were used to search for randomized controlled trials on the effects of different types of exercise on hypertension in middle-aged and older adults.

**Results:**

A total of 19 articles and 2,385 participants were included in the analysis. Aerobic exercise interventions [MD = −9.254, *P* < 0.05, 95% CI (−14.810, −3.698)] and static exercise interventions [MD = −10.465, *P* < 0.05, 95% CI (−18.135, −2.794)] had a significant effect on the improvement in systolic blood pressure (SBP). For diastolic blood pressure (DBP), aerobic exercise interventions [MD = −1.4096; *P* > 0.05, 95% CI (−8.2395, 5.4201)] and static exercise interventions [MD = −4.5206, *P* > 0.05, 95% CI (−14.0436, 5.0023)] were not statistically significant. The results of the surface under the cumulative ranking curve (SUCRA) showed that static exercise improved hypertension better than aerobic exercise.

**Conclusion:**

Aerobic exercise and static exercise have been shown to have a good effect on the improvement of hypertension, but the effect on DBP is not significant.

## 1. Introduction

The increasing prevalence of hypertension in modern times is a cause for worry. Hypertension is characterized by higher-than-normal blood pressure. The Seventh Report of the Joint National Committee on Prevention, Detection, Evaluation, and Treatment of Hypertension (2003 guidelines) stated that blood pressure varies throughout the day, and blood pressure measurements that are always higher than normal may indicate hypertension; the higher the blood pressure level, the greater the risk of other health problems, such as heart disease, heart attack, and stroke (1). The American Society of Cardiology/American Heart Association Guidelines for Prevention, Detection, Evaluation, and Management of Adult Hypertension (2017) indicate that normal blood pressure is below 120/80 mmHg; prehypertensive systolic blood pressure is 120–129 mmHg with diastolic blood pressure <80 mmHg; systolic blood pressure for hypertension is 130 mmHg or higher, and diastolic blood pressure is 80 mmHg or higher (2). The growth rate of the population affected by hypertension gradually increases with age, especially in middle-aged people. For adults aged 45 years without hypertension, the risk of hypertension over the next 40 years was 93% in African Americans, 92% in Hispanic individuals, 86% in white individuals, and 84% in Chinese adults (3). In the Framingham Heart Study, ~90% of all adults aged 55 or 65 years without hypertension developed hypertension during their lifetime (4). Hypertension usually develops over time. It may occur due to unhealthy lifestyle choices, such as insufficient physical exercise, excessive alcohol consumption, or an abnormal diet. Non-pharmacological therapy alone is particularly suitable for preventing hypertension and is used to manage the conditions of adults with hypertension or people with mild hypertension (5).

Non-pharmacological interventions can be performed through lifestyle-changing behavioral strategies and the promotion of physical activity, with the goal of modest BP reduction in the general population or more targeted BP reduction in adults at high risk of hypertension (6). The role of increased physical activity in reducing blood pressure has been repeatedly demonstrated in clinical trials, especially due to dynamic aerobic exercise and static isometric exercise (7, 8). Aerobic exercise is mainly fueled by aerobic metabolism, i.e., when the oxygen supply is sufficient. Generally, the exercise time is longer (more than 30 min), and the main muscle groups of the entire body contribute to the increased oxygen consumption during exercise (9). Quiet isometric stretching also has similar effects on functional improvement and therapeutic rehabilitation (10). Most trials were relatively short in duration, but increased physical activity has been a long-term intervention for lowering blood pressure and preventing hypertension (11). However, there is no main overview of the combined intervention of dynamic and static exercise modes, which needs further discussion and research. A meta-analysis revealed that interventions can prevent or improve the effects of hypertension and reported the effects of regular aerobic exercise on blood pressure, with an average reduction of 2–4 mmHg in SBP in adults with normal blood pressure and 5–8 mmHg in hypertensive patients (12). In addition, a meta-analysis showed that isometric exercise also had significant effects on lowering blood pressure (8). An isometric or static contraction is defined as a sustained muscle contraction (i.e., an increase in tension) with no change in the length of the involved muscle group (13). Isometric exercise might be an effective non-drug intervention for preventing and treating hypertension in older adults (14). There are a variety of active and passive stretches that are essential for the flexibility of older adults. This study mainly used network meta-analysis to explore the influence of different exercise types on hypertension in middle-aged and older adults and to explore the best exercise therapy to reduce hypertension in middle-aged and older adults.

## 2. Materials and methods

### 2.1. Inclusion criteria and literature search strategies

The literature search used the Boolean logic algorithm to obtain subject words and free words, searching databases including CNKI, Wanfang, Weipu, PubMed, Embase, Cochrane Library, and Web of Science for articles published up to 23 July 2023. The main subject words were “hypertension, blood pressure, high, high blood pressure, middle age, aerobic training, static training, combined training, and randomized controlled trial.” The literature screening applied PICOS strategies, which have been widely used in evidence-based medicine or practice. In the current study, the literature inclusion criteria were as follows: (1) subjects were middle-aged and older adults, with an average age of 45–64 years, without cardiovascular disease or other diseases; (2) randomized controlled experiments; (3) the exercise group only carried out the planned aerobic exercise or static stretching exercise, while the control group received no exercise intervention and no other intervention measures; (4) the intervention lasted more than 4 weeks; and (5) the aim of the study was mainly to compare the mean and standard deviation (SD) of resting blood pressure at baseline and at the end of the study between the control and exercise groups. The studies identified from the literature screening search were mainly those on the effect of exercise on blood pressure in middle-aged and older adults. Once those studies were identified, two investigators independently decided whether they should be included in the analysis. If the two authors disagreed, a third author intervened.

### 2.2. Data extraction and research quality evaluation

Data extraction was completed with a prespecified data extraction form that extracted relevant data and details related to the study's subject characteristics, interventions, and primary outcomes for systematic analysis. However, the usual PEDro score of two depends on how the study blinded the group and the treatment. In general, it is difficult to blind subjects in studies of exercise interventions. Therefore, these two points were excluded from the study analysis, and the PEDro assessment was based on eight terms, each scoring one point. The authors independently extracted and examined the data and assessed the quality of each study using the PEDro score.

### 2.3. Statistical analysis

Network meta-analysis was performed using Stata 16 software and RevMan 5.0 software. The outcome indicators selected were continuous variables, and the mean difference (MD) and 95% CI were used as effect size indicators. When *P* < 0.05, the inconsistent model was significant. These data cannot be directly analyzed by the consistency model; if *P* > 0.05, there was no significant difference between the direct and indirect comparisons, and the consistency model was used for analysis. A cumulative ranking probability map (SUCRA) was used to rank and compare the types of interventions. When SUCRA = 1, intervention measures are highly effective, while when SUCRA = 0, intervention measures are highly ineffective (15). According to the SUCRA values of the SBP and DBP indices, a stratified cluster analysis was used to explore the optimal treatment intervention.

## 3. Results

### 3.1. Literature screening process

**Figure 1:**
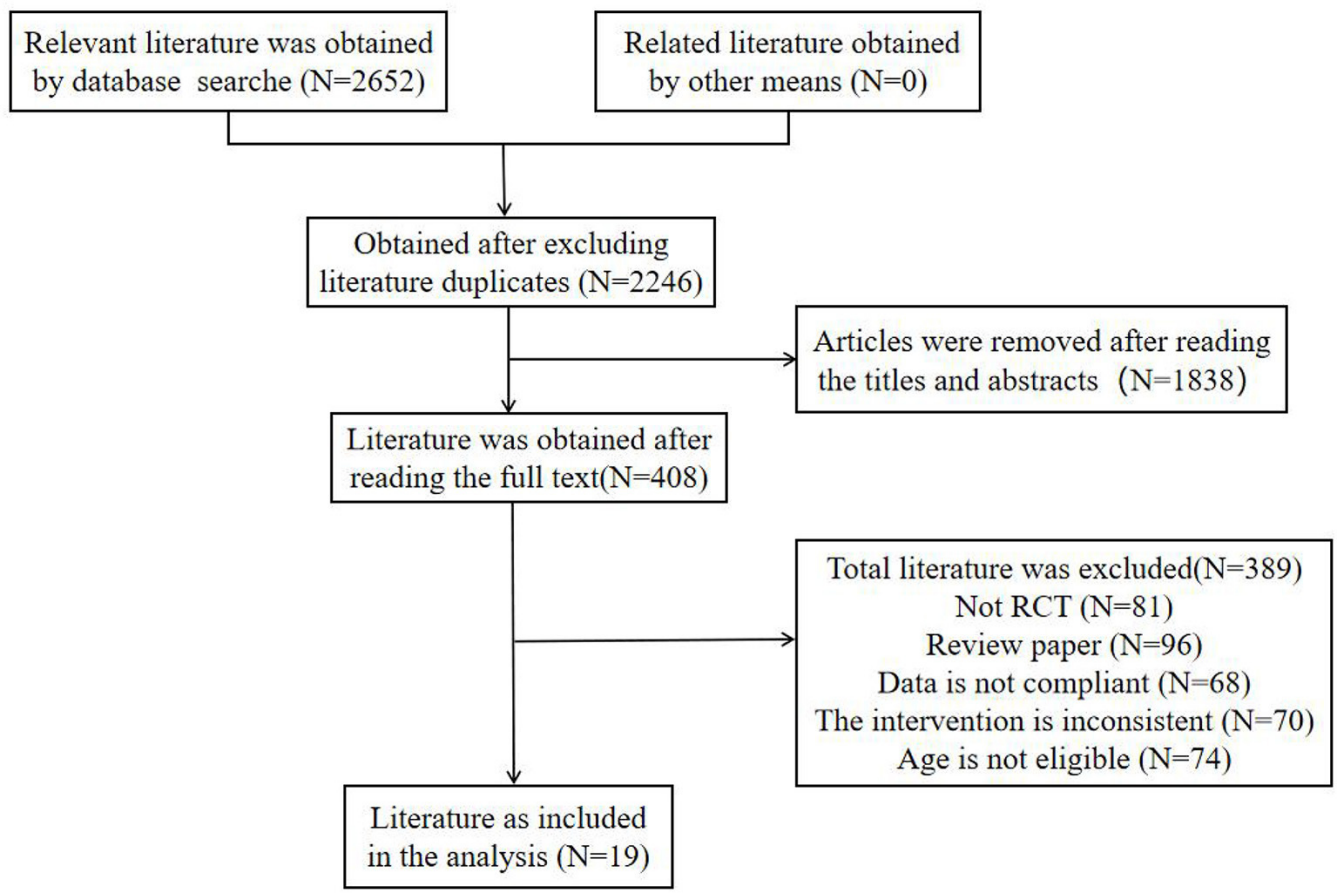
Flowchart of literature screening.

A total of 2,652 relevant articles were screened by a preliminary search of each database, and 19 articles, including 2,385 subjects, were finally included in the preliminary screening of titles and abstracts, as well as the rescreening of the full text. The main flow chart of the literature screening is shown in Figure 1.

### 3.2. The basic characteristics of the included studies and the evaluation of study quality

The 19 included studies mainly focused on hypertension in middle-aged and older adults. All subjects were grouped according to the principle of randomized control and the basic characteristics of the included studies (see Table 1).

**Table 1:** Basic characteristics of the included studies.

**References**	**Age (T/C)**	**Sample (T/C)**	**Intervention/control measures (T/C)**	**Intervention cycle**	**Outcome indicator**
Ko et al. (16)	61.9 ± 8.4/61.2 ± 15.0	20/20	ST/AT	8 weeks	SBP; DBP
Nemoto et al. (17)	62.3 ± 11.7/61.2 ± 13.3	27/26	ST/C	16 weeks	SBP; DBP
Lamina et al. (18)	58.27 ± 6.24	140/105	AT/C	8 weeks	SBP; DBP
Ng et al. (19)	50	30/30	AT/C	8 weeks	SBP; DBP
Cornelissen et al. (20)	59	26/22	AT/ST	10 weeks	SBP; DBP
Tsai et al. (21)	48.8 ± 6.3/49.3 ± 7.2	52/50	AT/C	10 weeks	SBP; DBP
He et al. (22)	58 ± 2/57 ± 2/58 ± 2	20/22/20	AT/ST/C	12 weeks	SBP; DBP
Zaleski et al. (23)	52.3 ± 10.8	12/12	AT/C	12 weeks	SBP; DBP
Wong and Figueroa (24)	57 ± 1/56 ± 1	14/14	ST/C	8 weeks	SBP; DBP
Dobrosielski et al. (25)	57 ± 6/56 ± 6	51/63	AT/C	6 months	SBP; DBP
Huiming and Qian (26)	55.7 ± 6.6/56.3 ± 7.5	39/41	AT/C	24 weeks	SBP; DBP
Jin-tao (27)	48.20 ± 7.0/49.62 ± 6.1	50/50	AT/C	3 months	SBP; DBP
Chen et al. (28)	45.77 ± 6.6/45.92 ± 7.2	48/48	AT/C	3 months	SBP; DBP
Hua et al. (29)	52.37 ± 10.45/51.90 ± 10.18	47/46	AT/C	6 months	SBP; DBP
Shengli (30)	50.48 ± 6.3/50.29 ± 6.1	61/61	AT/C	6 months	SBP; DBP
Guirong et al. (31)	50.5 ± 5.6	171/149/149	AT/ST/C	4 weeks	SBP; DBP
Siu et al. (32)	61.0 ± 5.7/62.2 ± 6.6/62.6 ± 6.2	181/181/181	AT/ST/C	12 weeks	SBP; DBP
Dos Santos et al. (33)	63.1 ± 2.3/64.2 ± 3.1/62.6 ± 2.5	20/20/20	AT/ST/C	16 weeks	SBP; DBP
Headley et al. (34)	58.0 ± 8.0; 57.1 ± 9.0	25/21	AT/C	16 weeks	SBP; DBP

All 19 included studies achieved “random assignment,” ITT intention-to-treat analysis, “statistical analysis between groups,” and “point measurement and variation measurement.” It is difficult to assess the overall quality of the study by blinding the subjects and therapists; therefore, these two points were excluded. The PEDro assessment is based on eight factors, and each is worth 1 point. Most PEDro scores were between five and six points, with an average score of ~6 points, and only one study reached a score of eight points. Overall, the results of the quality assessment of the literature were good (see Table 2).

**Table 2:** Quality evaluation of the included studies.

**Study**	**Random allocation**	**Distribute hide**	**Baseline similar**	**Subjects were blinded**	**Withdrawal rate <15%**	**ITT intentional treatment analysis**	**Statistical analysis between groups**	**Point measurements and variance magnitudes**	**Total**
Ko et al. (16)	1	0	1	0	1	1	1	1	6
Nemoto et al. (17)	1	0	1	0	1	1	1	1	6
Lamina et al. (18)	1	0	1	0	0	1	1	1	5
Ng et al. (19)	1	0	1	0	1	1	1	1	6
Cornelissen et al. (20)	1	0	0	0	1	1	1	1	5
Tsai et al. (21)	1	0	1	0	1	1	1	1	6
He et al. (22)	1	0	1	0	1	1	1	1	6
Zaleski et al. (23)	1	0	1	0	1	1	1	1	6
Wong and Figueroa (24)	1	0	1	0	0	1	1	1	5
Dobrosielski et al. (25)	1	0	1	0	1	1	1	1	6
Huiming and Qian (26)	1	0	1	0	1	1	1	1	8
Jin-tao (27)	1	1	1	0	1	1	1	1	6
Yan (28)	1	0	1	0	1	1	1	1	6
Hua et al. (29)	1	1	1	0	1	1	1	1	7
Shengli (30)	1	0	1	0	1	1	1	1	6
Guirong et al. (31)	1	0	1	0	1	1	1	1	6
Siu et al. (32)	1	1	1	0	0	1	1	1	6
Dos Santos et al. (33)	1	0	1	0	1	1	1	1	6
Headley et al. (34)	1	1	1	0	1	1	1	1	7

### 3.3. Consistency analysis results

The inconsistency model test of the 19 RCTs included showed that SBP (*P* = 0.228) and DBP (*P* = 0.667) were not significant. The results of the local inconsistency test by the node splitting method again resulted in *P* > 0.05, and the results of indirect comparison and direct comparison of the two indices were shown to be consistent, indicating that the consistency model should be used for analysis.

### 3.4. Results of network meta-analysis

**Figure 2:**
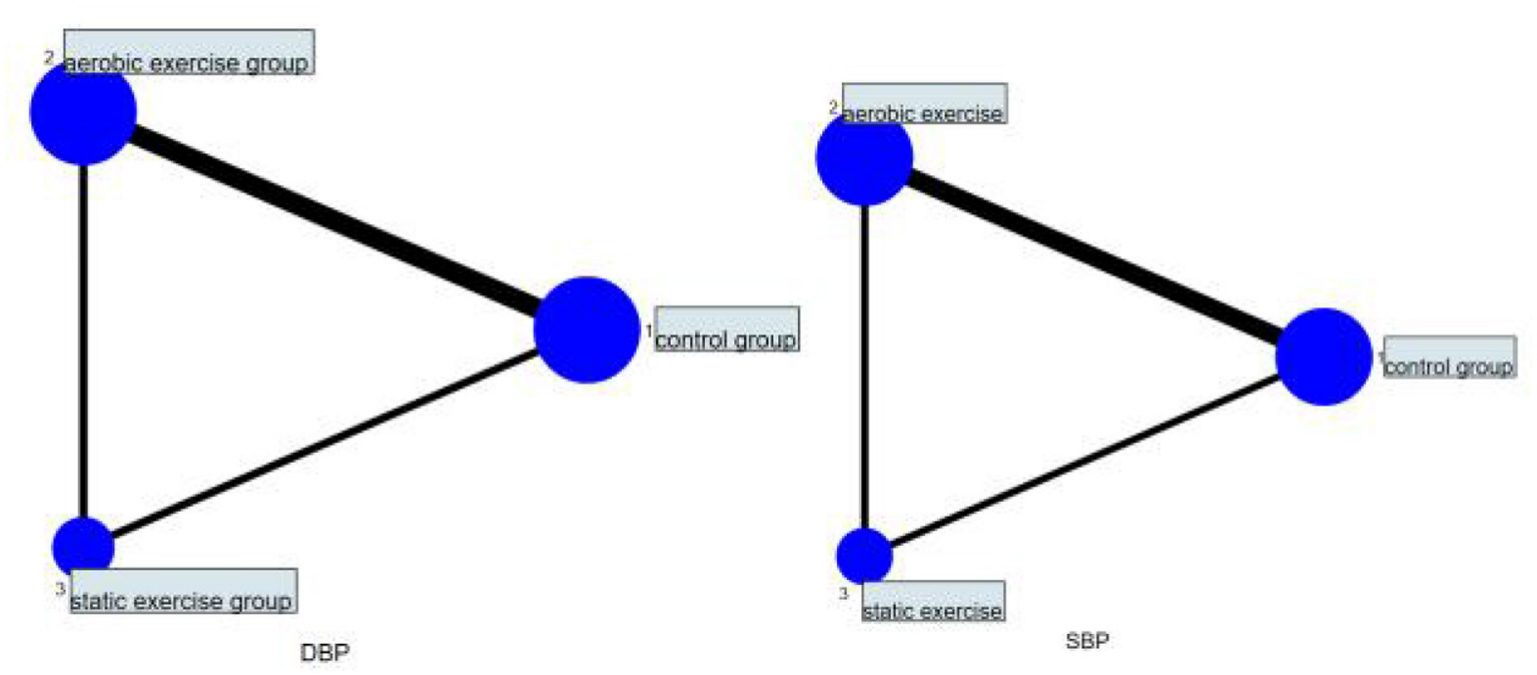
Network meta-analysis intervention diagram.

A total of 19 studies were included, including 11 studies comparing aerobic exercise with a control group, two studies comparing static exercise with a control group, and six studies comparing aerobic exercise with static exercise with the control group. For the network relationship between SBP and DBP of different exercise interventions, the circle area of aerobic exercise was the largest. The edge between aerobic exercise and conventional exercise was wider, indicating that studies comparing aerobic exercise with conventional exercise appeared most frequently among the included studies (Figure 2). Therefore, the effect of aerobic exercise on the hypertension of middle-aged and older adults is still supported by many studies, thus indicating a strong theoretical basis for its support.

### 3.5. Network meta-analysis of the effects of different exercise interventions on SBP

**Figure 3:**
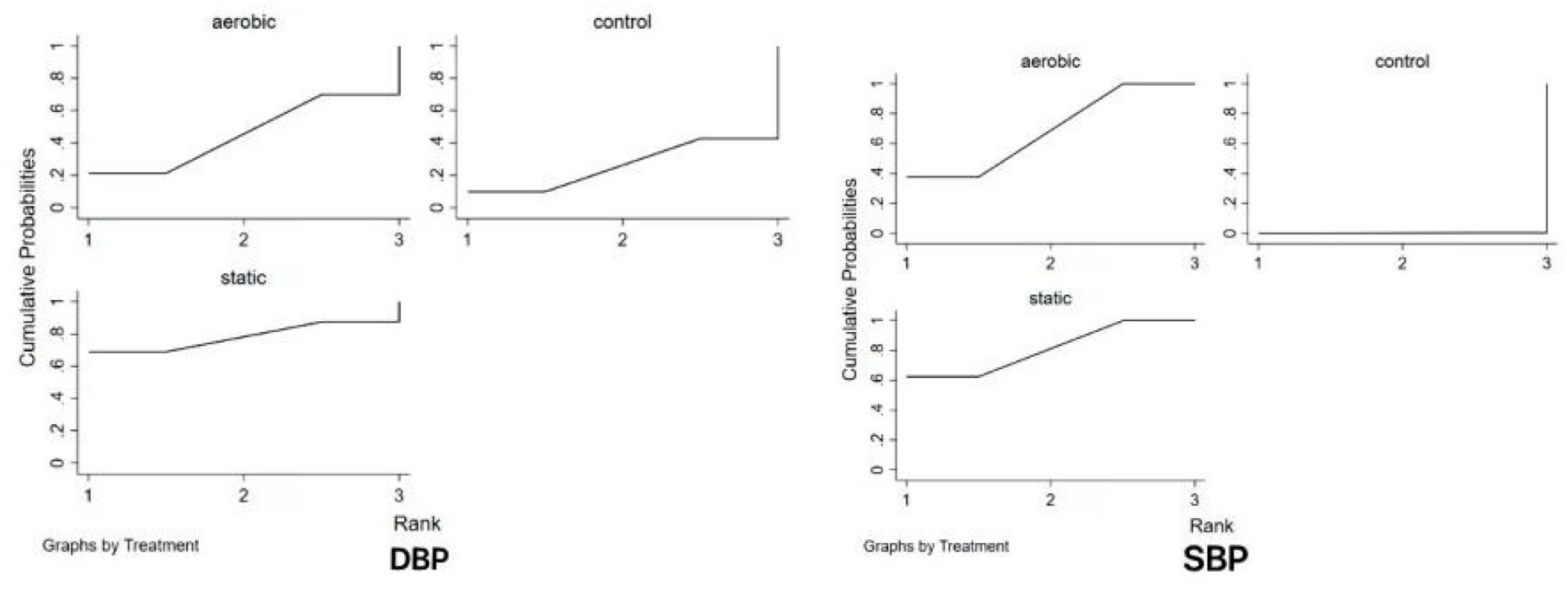
Probability ranking plot of different outcome measures of the intervention.

The results of the meta-analysis showed the MD value of aerobic exercise intervention [MD = −9.254, *P* < 0.05, 95% CI (−14.810, −3.698)] and the MD value of static exercise intervention [MD = −10.465, *P* < 0.05, 95% CI (−18.135, −18.135); −2.794] were better than those of the control group in improving SBP in hypertension. An indirect comparison between the two exercise modes showed no significant difference in SBP between the two groups (*P* > 0.05). The results of the SUCRA ranking showed that the effect of static exercise on SBP in hypertension was better than that of aerobic exercise (Figure 3).

### 3.6. Network meta-analysis of the effects of different exercise interventions on DBP

The results of the meta-analysis showed that the MD value in the aerobic exercise intervention [MD = −1.4096; *P* > 0.05, 95% CI (−8.2395, 5.4201)] and the MD value in the static exercise intervention [MD = −4.5206, *P* > 0.05, 95% CI (−14.0436, 5.0023)] had no significant effect on DBP improvement in hypertension (all *P* > 0.05). An indirect comparison between the two showed no significant difference in exercise mode between the two groups (*P* > 0.05). The results of the SUCRA ranking showed that static exercise had a better effect on DBP in hypertensive individuals (see Figure 3).

### 3.7. Detection of publication bias

**Figure 4:**
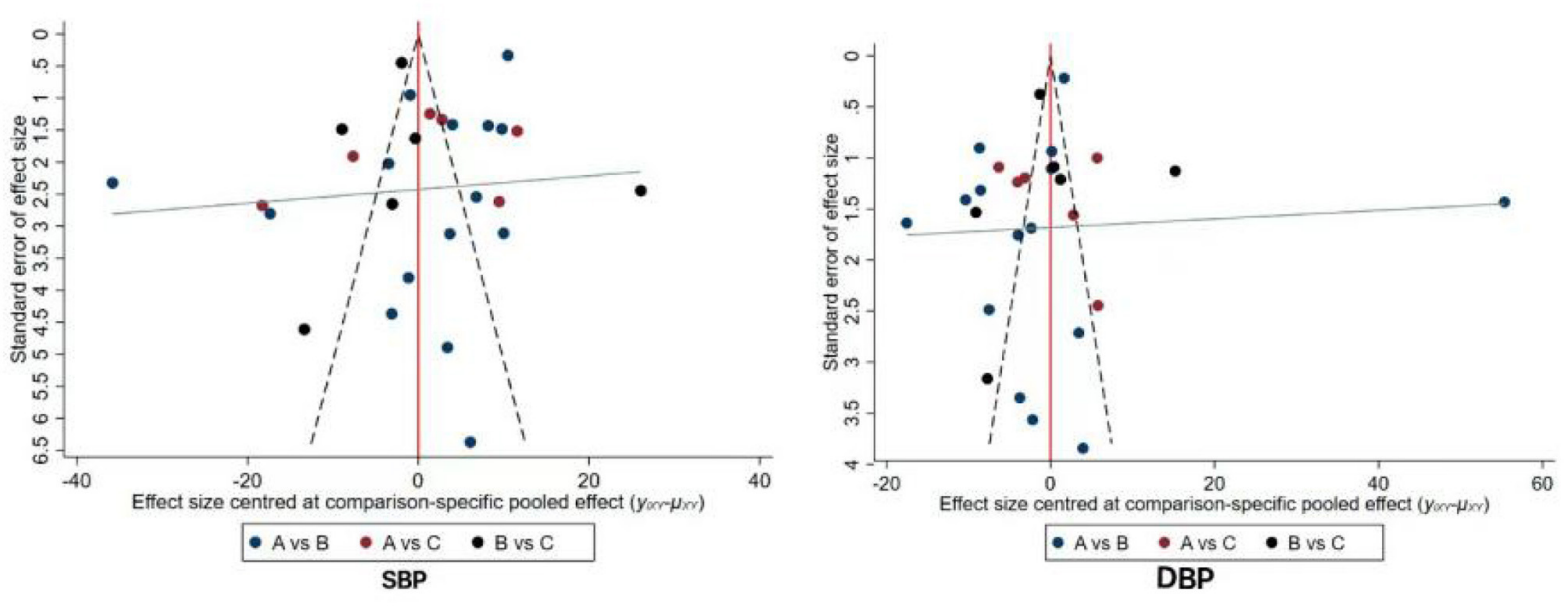
Funnel plots of publication bias for outcome measures of included studies. A: control group; B: aerobic exercise; C: static exercise.

Compared with the corrected funnel plot, the inverted funnel plot was symmetric, and the scattered points were distributed within the range of the inverted funnel plot (see Figure 4), indicating that there was little possibility of a small-sample effect or publication bias.

## 4. Discussion

### 4.1. Intervention effects of aerobic exercise on hypertension in middle-aged and older adults

Many recent studies have found that exercise modes such as isokinetic resistance training, limb stretching exercises, swimming, and running can reduce blood pressure (12, 35). Some studies have shown that aerobic exercise training can lower blood pressure by 5–7 mmHg (36). In this study, aerobic exercise was also found to have a significant effect on lowering SBP in middle-aged and older adults with hypertension, which can reduce the incidence of hypertension to some extent. Engaging in physical activity can also further prevent cardiopulmonary impairment and lower blood pressure (37). The incidence of hypertension increases with age, and the older the individuals are, the more their exercise ability will be affected, and the exercise mode or intensity will be limited (38). The incidence of many chronic diseases, such as cardiovascular disease and hypertension, further increases with age (39, 40). Many forms of aerobic exercise can lower blood pressure in middle-aged and older adults, such as jogging, square dancing, and aerobics, all of which have good effects. Whelton et al. found that aerobic exercise had an overall net effect of reducing SBP and DBP by 3.84 and 2.58 mmHg, respectively (7). Hagberg et al.'s review showed that in hypertensive adults aged 60 or older, SBP and DBP values were reduced by an average of 7.6/8.8 mmHg (41). In this study, aerobic exercise had a significant effect on SBP but not on DBP. This suggests that in this study, aerobic exercise effectively lowered blood pressure in middle-aged and older adults with hypertension, which has certain clinical significance and value for the health and medical conditions of both middle-aged and older adults.

### 4.2. Isometric exercise intervention effects on hypertension in middle-aged and older adults

For middle-aged and older individuals, the most common isometric exercises are static stretching exercises, which are primarily defined as sustained muscle contraction (i.e., increased tension) without changes in the length of the involved muscle groups. To date, relatively little attention has been given to the effects of isometric exercise training on resting blood pressure. The results from previous meta-analysis suggest that although the overall sample size is still small, isometric exercise training may produce greater reductions in resting blood pressure (42, 43). The study results also showed that the improvement effect of isometric exercise on the SBP and DBP of middle-aged and older hypertensive individuals was better than that of aerobic exercise on the SBP and DBP of hypertensive individuals, which is similar to the results of the previous meta-analysis. However, middle-aged and older individuals should not excessively perform high-load isometric exercises, as improper training may put pressure on the heart, potentially leading to other diseases. Since isometric exercise is not traditionally recommended for hypertensive subjects, isometric training is generally only applicable to some specific exercise programs. However, small-scale, short-term studies conducted in subjects with normal blood pressure and hypertensive subjects suggest that performing 3–4 short isometric exercises per week can lower systolic and diastolic blood pressure (44). Isometric exercise also has effects on lowering blood pressure in middle-aged and older adults, but its advantages compared to dynamic exercise training (aerobic exercise) may only be limited to blood pressure, and further investigation is still needed regarding other possible aspects. Additionally, middle-aged and older adults need to perform these exercises within their capacity to avoid injuries to the joints and other body parts.

### 4.3. The impact of different exercise modes on hypertension in middle-aged and older adults

The results of the included literature showed that different exercise intervention modes significantly lower blood pressure in middle-aged and older adults. Aerobic exercise modes such as slow running, hiking, cycling, and brisk walking, as well as static exercises such as stretching, isometric exercises, and combined exercises with low-intensity dynamic and static loading, all have an impact on lowering hypertension in middle-aged and older adults. Currently, international treatment guidelines for primary and secondary prevention of hypertension in China generally recommend lifestyle changes (quitting smoking, weight loss, exercise training, healthy eating, and reducing sodium intake) as the first-line treatment (45). Non-pharmacological treatments can promote the reduction and improvement of blood pressure in middle-aged and older adults, contributing to their physical and mental health development. In daily life, older adults need to engage in reasonable and healthy exercises to cope with the diseases caused by hypertension and provide a suitable and comfortable living environment.

## 5. Limitations

This study has several limitations. First, it only includes aerobic and static exercise methods, while the effects of other exercise modalities, such as dynamic resistance training, have not been considered. Second, due to the limited number of studies included, there may be a certain degree of selection bias present. Finally, the study incorporates various outcome assessment indicators, and the results of the heterogeneity test suggest that the heterogeneity is relatively high. Therefore, the quality control standards for future clinical trials should be informed by evidence-based medicine standards.

## 6. Conclusion

Both aerobic exercise and static exercise have significant effects on the reduction of blood pressure in middle-aged and older adults. The effect of both exercise modes on systolic blood pressure, but not diastolic blood pressure, is significant. According to the results of the SUCRA ranking, static exercise is better than aerobic exercise in reducing blood pressure in middle-aged and older adults. Appropriate exercise is effective for middle-aged and older adults with hypertension and has certain benefits in reducing hypertension.

## Data availability statement

The original contributions presented in the study are included in the article/supplementary material, further inquiries can be directed to the corresponding author.

## Author contributions

WG wrote the article. ML was responsible for data collection and analysis. TH checked and revised the manuscript. All authors contributed to the article and approved the submitted version.
